# Medical Items

**Published:** 1893-09

**Authors:** 


### *MEDICAL ITEMS.*

---

Dr. James Camak, of Athens, died on the 13th of August, in the sixty-eighth year of his age. He had been a resident of Athens for more than sixty years.

---

The International Medical Congress, which was to meet at Rome on the 24th inst., has been postponed until April, 1894, on account of the cholera prevailing in Italy.

---

Dr. A. G. Hobbs will attend the Pan-American Medical Congress and read a paper on "The Simplest and Most Practical Electric Apparatus Especially for Galvano-Cautery Operations."

---

In the list of delegates to the Pan-American Medical Congress, published last month, the name of Dr. W. D. Travis, of Covington, Ga., was unintentionally omitted.

---

Dr. J. Y. Hopkins, of Thomasville, has been spending some time at Colorado Springs, and it is gratifying to The Journal to be able to report that he is greatly improved in health.

---

The medical world will learn with much regret of the death of Professor J. M. Charcot, the great neurologist of Paris, and probably the foremost neurologist in the world. Dr. Charcot was in his sixty-eighth year.

---

Before the Colorado board of medical examiners hereafter no diploma will be recognized as entitling the holder to pratice medicine which is not issued by a school requiring three years of study, and certain preliminary qualifications. In the absence of such diploma the applicant must pass the usual examination.

THE AMERICAN ELECTRO-THERAPEUTIC ASSOCIATION will hold its third annual meeting in Chicago on the 12th, 13th and 14th instants. The two main subjects for discussion will be, (1) "What are the Possibilities of Electricity in the Treatment of Fibroid Growths," and (2) "The Influence of Frequency of Interruptions and Character of Induced Current Waves upon Physiological Effect." Among the foreign therapeutists who have a place on the program are Apostoli and Gautier of Paris, DuBois-Reymond of Berlin, La Torre of Rome, Newman Lawrence, of London, and Lapthorn Smith of Montreal.

---

DR. PERCY N. DE DUBOEAY is a physician at Tallulah Falls. He is also a Count. In May he began the Keeley treatment at Indian Springs, and was attended by Dr. Harris Hall. Dr. Hall gave him the usual hypodermics, and several abscesses formed on the Count's left thigh, which finally resulted in paralysis of the limb. Dr. De Duboeay further says that Dr. Hall was a drinker himself and that on one occasion he fell against him in such a way as to cause him to sustain a fracture of the leg. For all of which damages, abscesses and fracture of leg, with loss of function, Count De Duboeay now sues the Keeley Company for fifteen thousand dollars. The case is rather novel and will be watched with interest.

---

A NEW edition of Dunglison's Medical Dictionary is announced as in press for early publication. It has been thoroughly revised and greatly enlarged, and will contain about forty-four thousand new medical words and phrases. Pronunciation has been introduced into the new edition by means of a simple phonetic spelling. This work has always been noted for the fullness of its definitions, ample explanation being its distinguishing characteristic. In the new edition much encyclopedic information, difficult of access elsewhere, will be found conveniently at hand. Especial attention has been devoted to matters of practical value. A review will appear in an early issue.

